# Thrombocytopenia Occurrence With NxStage vs. Prismaflex Continuous Renal Replacement Systems

**DOI:** 10.7759/cureus.99659

**Published:** 2025-12-19

**Authors:** Reza Khorsan

**Affiliations:** 1 Medicine/Nephrology, David Geffen School of Medicine, University of California Los Angeles, Los Angeles, USA

**Keywords:** continuous renal replacement therapy (crrt), dialysis, nxstage system one, prismaflex, thrombocytopenia

## Abstract

In this article, we will present a case of a 64-year-old male presenting with severe renal failure requiring the initiation of continuous renal replacement therapy (CRRT) with the NxStage system (NxStage Medical Inc., Lawrence, USA). He develops significant thrombocytopenia less than 48 hours after starting CRRT with NxStage. After switching to the Prismaflex CRRT system (Baxter Healthcare Corporation, Deerfield, USA), his serum platelet values started to increase towards normal. It is believed that the NxStage system is more commonly associated with thrombocytopenia than the Prismaflex system. The exact reason is not known. It is paramount that practitioners of critical care medicine are aware of this complication.

## Introduction

Nephrologists and intensivists are familiar with the complication of thrombocytopenia in the intensive care unit (ICU) population. It is a common complication of critical illness [1]. Thrombocytopenia is common in patients undergoing hemodialysis, especially continuous renal replacement therapy (CRRT) [2]. The causes for thrombocytopenia are due to a variety of reasons, and the patients’ blood contact with the dialysis membrane itself is thought to play a crucial role in the development of CRRT induced thrombocytopenia [1-5]. Differences in dialysis membranes due to various materials used to manufacture the fibers and the sterilization techniques used in the manufacturing process may cause platelet activation and consumption [6]. Common CRRT systems used in the ICU include the NxStage system (NxStage Medical Inc., Lawrence, USA) and the Prismaflex system (Baxter Healthcare Corporation, Deerfield, USA). Although there are reports of thrombocytopenia with both systems, the NxStage system may be more implicated with this complication. The exact reason for this has been postulated to be related to the materials used for the dialysis membrane or the sterilization process.  

## Case presentation

**Figure 1:**
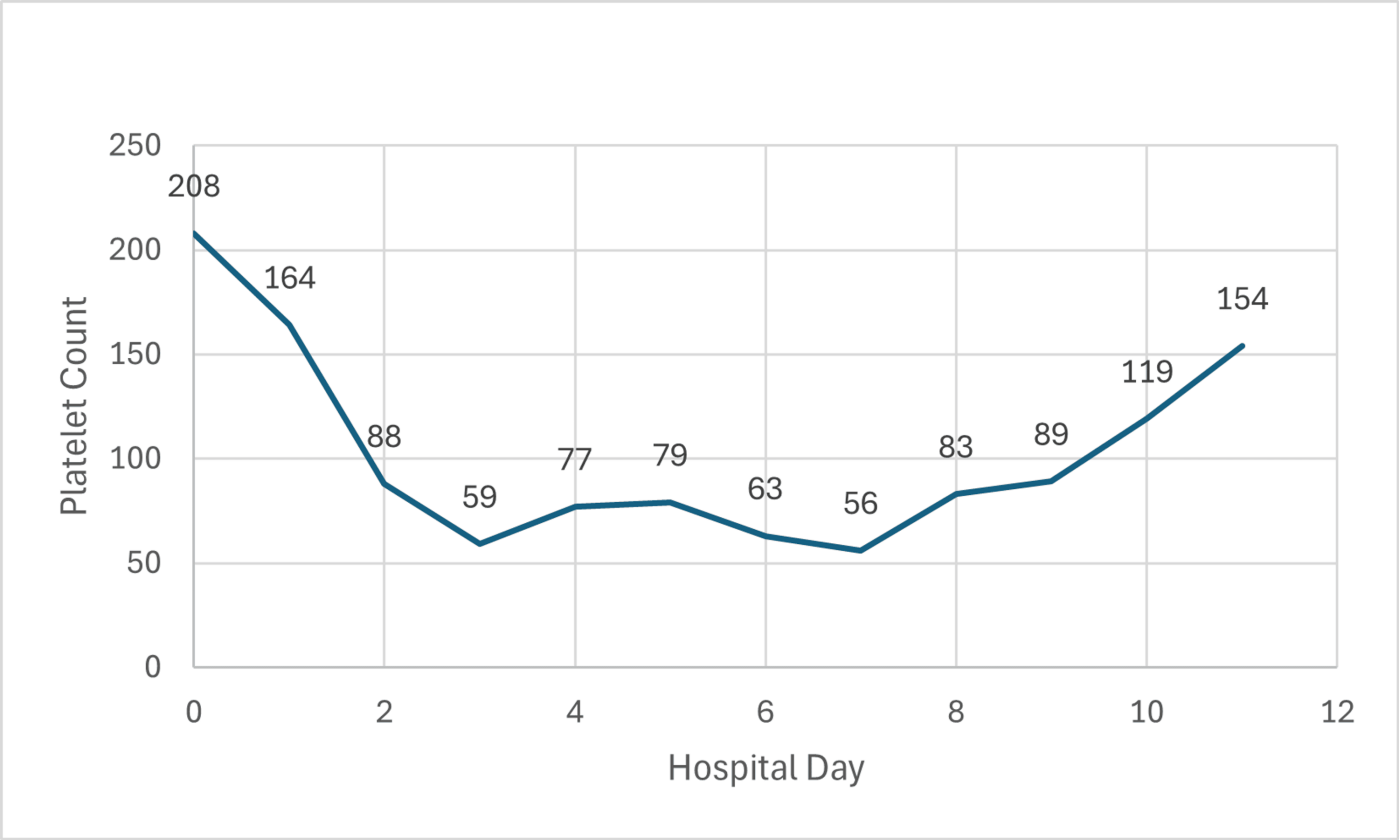
Platelet Count v Hospital Day

A 64-year-old male with a past medical history of diabetes mellitus type 2 (DM2) who takes metformin and glimepiride presented to the emergency department with symptoms of shortness of breath for one week. He had been in his usual state of health on a trip abroad. He experienced progressive shortness of breath, without cough or fever. He started to experience orthopnea symptoms and headed back home to the United States. His symptoms progressed, and he decided to seek emergency care. Upon initial encounter, he was noted to be tachycardic to 152 bpm and hypoxic, saturating 90% on room air. ECG showed atrial flutter. Chest x-ray in the ED showed a large right pleural effusion with a possible mass. A CT angiogram was obtained, which showed bilateral pleural effusions, no evidence of pulmonary embolism, diffuse nodular interlobular thickening suspicious for lymphangitic carcinomatosis, and ground-glass opacities suspicious for infection. His initial laboratory values are in Table 1 below. He was started on broad-spectrum antibiotics and admitted to the ICU on a heparin drip. The following morning, he had a cardiac arrest. He was resuscitated and quickly became anuric, with worsening anion gap metabolic acidosis due to diabetic ketoacidosis. Laboratory data post return of spontaneous circulation are in Table 1 below. He was promptly started on NxStage continuous renal replacement therapy. The following morning, his platelets dropped to 88,000 per uL, with a nadir 24 hours later to 59,000 per uL. He was then switched to the Prismaflex continuous renal replacement system, and his platelet levels increased daily till reaching 154,000 per uL seven days after the switch. Please see Figure 1 below. A heparin-induced thrombocytopenia (HIT) antibody panel was sent when the platelets started to decrease, and resulted in negative results a few days later. He was on CRRT for a total of 10 days and then transitioned to intermittent hemodialysis, which he required at the time of discharge from the hospital. 

**Table 1:** Laboratory Values on Presentation and After Cardiac Arrest ul=microliter; mg=milligrams; dl=deciliter

Initial clinical laboratory values	Results on presentation	Results after cardiac arrest	Reference
White Blood Cell Count	7500 cells per ul	13,300 per ul	4200-9950 cells per ul
Hemoglobin	14 g per dl	12.1 g per dl	13.5- 17.1 g per dl
Platelets	208,000 per ul	164,000 per ul	143,000-398,000 cells per ul
Serum Creatinine	1.1 mg per dl	1.8 mg per dl	0.6-1.3 mg per dl

## Discussion

Thrombocytopenia is common in hemodialysis patients and critically ill patients undergoing CRRT in ICUs. The causes are numerous and include various patient-related factors as well as non-patient-related factors [1]. Common patient-related factors include acute illness, organ dysfunction, bacteremia, viremia, and severe sepsis. Non-patient-related factors include medications and exposure to tubing and various intravascular devices [1,2]. A common cause of thrombocytopenia in these patients is the use of continuous renal replacement therapy, CRRT, with some estimates as high as 50% [3,4].

Thrombocytopenia due to dialyzer membranes is a well-known complication of hemodialysis [5]. Older cuprophane and cellulose membranes were more frequently implicated due to complement activation [6]. Newer polysulfone dialyzers are more biocompatible, but they can still cause thrombocytopenia. It is thought that the sterilization techniques used to manufacture the dialyzer may be the cause of complement activation and thrombocytopenia, with electron-beam sterilized membranes causing thrombocytopenia more often than gamma sterilized membranes [7]. However, there are case reports that suggest the type of polysulfone membrane used is more contributory, rather than the sterilization technique utilized [8].

The NxStage CRRT systems use a gamma-sterilized polysulfone-based dialyzer in a closed-circuit kit system. Although this type of dialyzer is supposed to be more biocompatible, there are case reports of thrombocytopenia with its use [9,10]. In one case report, two patients developed thrombocytopenia after switching from conventional hemodialysis to home NxStage dialysis. The thrombocytopenia resolved when they went back to conventional in-center hemodialysis and recurred in the one patient who retried NxStage-based home hemodialysis [11]. It is important to know that this was in patients on the NxStage home hemodialysis system and not CRRT, but the dialyzers are similar. The Prismaflex system utilizes a non-polysulfone dialyzer, specifically the AN69 ST hollow fiber membrane. It is a modified cellulose membrane made of polyarylethylsulfone. It is sterilized using ethylene oxide, which is a gas sterilization method. Although there have been studies that evaluated thrombocytopenia in critically ill patients undergoing CRRT that included patients on the Prismaflex system [2], to date, there have been no specific published case reports of thrombocytopenia induced definitively by the Prismaflex CRRT system. 

It has been unknown whether the NxStage system more frequently causes thrombocytopenia and to a more significant degree than the Prismaflex system. A recent study by Brumit et al. shows that it may [12]. In this retrospective cohort study evaluating thrombocytopenia in patients on NxStage vs. Prismaflex in a cardiac ICU in an academic hospital, NxStage had an adjusted odds ratio greater than 5.5 for thrombocytopenia compared with Prismaflex. The exact reason for this is unknown, and it is probably multifactorial. It is possible that it may be due to the sterilization technique of the dialyzer, or it could be more inherent to the fibers of the dialyzer itself. Another possibility could be due to the slight differences in the modalities of dialysis itself, continuous venovenous hemodialysis (CVVHD) in the NxStage system, and continuous venovenous hemodiafiltration (CVVHDF) in the Prismaflex system. Regardless, thrombocytopenia happens more frequently and is more pronounced with the NxStage system compared to the Prismaflex system in this study [12]. 

## Conclusions

NxStage may be associated with thrombocytopenia more often than Prismaflex. The exact reason is unknown and needs to be further studied. Large prospective studies would be helpful. It is prudent for nephrologists and intensivists to be aware that thrombocytopenia may occur with all CRRT systems. If it occurs in patients on the NxStage system, switching to Prismaflex system may prevent unnecessary HIT testing and stoppage of CRRT therapies in these patients.
